# The Late Surgeon-General Sir William James Moore, K.C.I.E.

**Published:** 1896-09-19

**Authors:** 


### THE LATE SURGEON-GENERAL SIR WILLIAM JAMES MOORE, K.C.I.E.

Sir William Moore who was honorary physician to the Queen and to the Viceroy of India, died at his residence, 15, Portland Place, on September 9th, at the age of 68. He was a son of Mr. Edward Moore, D.L., of Halesowen, Worcestershire, and received his medical education at Queen's College, Birmingham, where he was a most industrious student, and carried off many prizes. He was subsequently, for three years, resident surgeon to the Queen's Hospital in that city, and, whilst occupying that post, in 1852, he obtained a prize, given by the British Medical Association, for a "series of surgical cases," the subject being "the treatment of the contraction of the cicatrices of burns." In the same year he entered the Indian Medical Service, in which he filled many important posts. He served in the Persian War of 1856-7, was surgeon to the Rajpootana Political Agency, and Superintendent of Dispensaries and of Vaccination in Rajpootana. In 1882 he was Dean of the Faculty of the University of Bombay. He served for five years as Deputy Surgeon-General, and for nearly the same length of time as Surgeon-General. Whilst Surgeon-Major in 1873 he gained a prize of 1,000 rupees, offered by the Government of India, for the best manuscript of a "Manual of Family Medicine and Hygiene for India," for which it was understood that upwards of sixty essays were sent in. This was published for the Government, and has gone through more than six editions. His success in this respect no doubt brought him into notice and led to his promotion to the higher ranks. He was also the author of a work on "The Immediate Treatment of Accidents and Injuries," which was published by the Government of Bombay. He was an indefatigable writer, both of books and in the medical and lay press. Among his best-known works are, "A Manual of the Diseases of India," "Health in the Tropics; or, Sanitary Art Applied to Europeans in India," "Health Resorts for Tropical Invalids," &c. He was great on the opium question, his pamphlet, "The Other Side of the Opium Question," taking very strongly the line that the consumption of the drug was not nearly so injurious as its opponents made out, whilst in many instances it was beneficial. On his retirement he was knighted, and the medical officers of the Bombay Presidency subscribed to present him with his portrait—life-size, in uniform—which hangs in the Town Hall of Bombay. Of this testimonial of the esteem of those who had been under his command he was justly very proud. He leaves a widow and one son, Captain Arthur Trevelyan Moore, R.E.. His brother, Mr. Thomas Moore, F.R.C.S., who was formerly surgeon to the Petersfield Cottage Hospital, and who is at present surgeon to the Miller Memorial Hospital, Greenwich, has for years been in practice at Lee. Sir William Moore was one of the earliest contributors to THE HOSPITAL, and at one time wrote largely for it on medical subjects. He was an indefatigable worker, and a singularly clear thinker of marked capacity and shrewdness. Companionable, loyal, and obliging as a colleague, he was ever resourceful and ready, whilst his friendship once bestowed was never broken.

